# Mathematical model and nanoindentation properties of the claws of *Cyrtotrachelus buqueti* Guer (Coleoptera: Curculionidae)

**DOI:** 10.1049/nbt2.12089

**Published:** 2022-05-26

**Authors:** Longhai Li, Wei Sun, Ce Guo, Huafeng Guo, Liu Lili, Ping Yu

**Affiliations:** ^1^ School of Mechanical and Electrical Engineering Xuzhou University of Technology Xuzhou China; ^2^ Institute of Bio‐inspired Structure and Surface Engineering College of Mechanical and Electrical Engineering Nanjing University of Aeronautics and Astronautics Nanjing China

**Keywords:** beetle claw, mathematical model, mechanical properties, micro‐structures, nanoindentation

## Abstract

Scanning electron microscopy (SEM) was used to observe the macroscopic, microscopic, and cross‐sectional structures of the claws of *Cyrtotrachelus buqueti* Guer (Coleoptera: Curculionidae), and a mathematical model of a claw was used to investigate the structure–function relationships. To improve the quality of the SEM images, a non‐local means (NLM) algorithm and an improved NLM algorithm were applied. After comparison and analysis of five classical edge‐detection algorithms, the boundaries of the structural features of the claw were extracted based on a B‐spline wavelet algorithm, and the results showed that the variable curvature of the beetle claw enhances its adhesion force and improves its strength. Adhesion models of the claw were established, and the mechanical properties of its biomaterials were measured using nanoindentation. Considering that the presence of water can affect the hardness and Young's modulus, both ‘dry’ and ‘wet’ samples were examined. For the dry samples, the hardness and Young's modulus were 0.197 ± 0.074 GPa and 1.105 ± 0.197 GPa, respectively, whereas the respective values for the wet samples were both lower at 0.071 ± 0.030 GPa and 0.693 ± 0.163 GPa. This study provides data that can inform the design of climbing robots.

## 1 INTRODUCTION

An important approach to exploring nature and the complex interactions of all organisms is (i) understanding a wide range of biological materials and structures with specific properties matched to function and (ii) demonstrating how selective pressure shaped the evolution of animal locomotion [1, 2, 3, 4]. Insects constitute the largest family of known organisms with ∼62,000 species worldwide and accounting for almost three‐quarters of all living things on Earth [5, 6], and among insects, weevils (Coleoptera: Curculionidae) are one of the most species‐rich groups of organisms on the planet [7, 8]. One of the main reasons for the success of insects is their locomotion by which they can walk to find food and avoid natural enemies [9, 10, 11].

Locomotion is a fundamental skill for the survival of the majority of animals, and it is also the source of life [12, 13, 14, 15]. Natural evolution is always accompanied by changes in locomotion, even in the process of plant growth, which is still inseparable from motion and is simply a passive form of motion or implicit internal motion. To adapt to various harsh environments and their complicated requirements, insects had to develop crawling abilities in the process of their independent evolution [16]. Because of the different characteristics of different environments, the crawling and attachment patterns of insects are varied. Climbing creatures can be divided broadly into three categories according to these patterns: (i) those with soft smooth pads (e.g., tree frogs, flies, and ants) [17, 18, 19, 20], (ii) those with hairy pads (e.g., geckos, mosquitoes; also, some spiders have many very fine hairs that achieve adhesion via van der Waals forces on almost any surface) [21, 22], and (iii) those with claws. However, hairy and soft smooth pads have specific requirements for their contact surfaces, whereas claws are more suitable for various rough and irregular surfaces [23, 24]. This is why most insects that can crawl on vertical surfaces or in extreme conditions have the latter configuration.

For adaptation to rough vertical and inclined surfaces, biologists have attempted to develop wall‐climbing robots by drawing inspiration from the claws of some climbing animals. One study reported the manufacture of biomimetic toes based on the structure of the foreclaws of the mole rat (*Scaptochirus moschatus*), the toes being created using reverse engineering and rapid prototyping [25]. The results of that study indicated that the toe geometry has a significant effect on its soil‐cutting performance; it reduces soil‐cutting resistance and improves the designs of soil‐cutting components, resulting in reduced energy and increased efficiency. The authors in reference [26] used a physical model to reveal the laws governing the interaction between the claws and rough surfaces, and they designed a four‐legged robot based on these claws with the ability to climb a rough vertical surface; this robot was found to have high stability and adaptability while climbing on vertical rough surfaces up to a speed of 4.6 cm/s.

Mimicking the sharp directional spines on the legs of some insects, microspines use one or more small steel hooks to grasp asperities (e.g., pits and ledges) on a rough surface to provide adhesion for a robot, allowing it to climb. Microspine robots have been found to be able to traverse varied terrains, including painted curbs, stairs with overhanging face angles, and rough vertical walls [27]. Another study reported the design of an insect‐inspired gravity‐independent mobility and drilling robot for natural rock surfaces that use a hierarchical array of claws [28]; it was noted that the robot would be able to grip rocks on the surfaces of asteroids and comets as well as the cliff faces and lava tubes of Mars.

*Cyrtotrachelus buqueti* Guer is classified in the phylum Arthropoda, class Insecta, order Coleoptera, and family Curculionidae [29, 30], and its claws of the weevil are successful products of evolution. In previous work, we subjected to the adhesion force of a claw of this species to approximate testing using a one‐dimensional force sensor, and we found that the force can reach 2–5 N. However, the testing weevil weighed only 1–3 g, meaning that when a claw was attached to an appropriate surface, the maximum adhesion force could reach at least 67 times its own weight. As is well known, a strong horse weighing 700 kg can pull only 3.5 tons of cargo on good roads, which is only five times its weight; humans can also pull approximately five times their own weight. We have noted previously that the adhesion force of a claw of *C. buqueti* Guer relates to external conditions, such as the diameter of a bamboo shoot, the shape of a surface, and the surface roughness; however, we reason that it is also closely related to the geometric features of the claw (such as its curvature and concavity or convexity) and its mechanical properties.

With the above observations in mind, in the present study, we built a mathematical model of a foreclaw, investigated its structure–function relationships, and examined its nanomechanical properties. Our primary aim was to provide some basic data or a biological template for developing wall‐climbing robots and other crawling devices.

## 2 MATERIALS AND METHODS

### 2.1 Microscopy examination

*Cyrtotrachelus buqueti* Guer is an incredibly diverse species of weevil found mostly in southern China but also elsewhere in the world. The weevils for the present study were obtained from Leshan in Sichuan Province in China. They were adult weevils (mass: 1837 ± 26 mg, body length: 36.2 ± 4.3 mm, body width: 12.6 ± 2.2 mm, *N* = 16), of which ten were used in the scanning electron microscopy (SEM) imaging and six were used in the nanoindentation experiments. For SEM examination, the specimens were first anesthetized with CO_2_, then their claws were dissected from their forefeet using a scalpel and a pair of tweezers. The claws were cleaned with pure water, dehydrated in a series of increasing ethanol concentrations (50%, 70%, 80%, and 90%), and then critical‐point dried. Finally, the freeze‐dried claws were sputter‐coated with gold–palladium alloy and then imaged using a scanning electron microscope (Quanta 200; FEI, USA) operated with an accelerating voltage of 20 kV.

### 2.2 Improved non‐local means (NLM) algorithm

To explore the structure–function relationships of the weevil claws, feature‐curve models were investigated. Because of the original images of the claws appearing blurred or containing ‘snowflake’ noise, both the non‐local means (NLM) method and an improved NLM method were used to reduce noise while preserving the image details, thereby improving the overall image quality [31, 32].

Introduced by Buades et al., the NLM algorithm has a proven ability to reduce noise while preserving structures and details by taking advantage of the structural self‐similarity properties of an image [29, 33]. It estimates pixel values by using a weighted average of pixel intensities in an image, the mathematical principles of which can be expressed as

(1) $$NL\left[v\right]\left(i\right)=\frac{1}{C\left(i\right)}\sum_{j\in I}w\left(i,j\right)v\left(j\right),$$

(2) $$w\left(i,j\right)=\frac{1}{C\left(i\right)}\exp\left(-\;\frac{{\left\|v\left(N\left(i\right)\right)-v\left(N\left(\;j\right)\right)\right\|}_{2,\alpha }^{2}}{h^{2}}\right),$$

(3) $$C\left(i\right)=\sum_{j\in I}\exp\left(-\;\frac{{\left\|v\left(N\left(i\right)\right)-v\left(N\left(\;j\right)\right)\right\|}_{2,\alpha }^{2}}{h^{2}}\right),$$

where *i* is a pixel, *I* is the image domain, w(i,j) is the similarity weight coefficient between two patches *N*(*i*) and *N*(*j*) centred around pixels *i* and *j*, ${\left\|v\left(N\left(i\right)\right)-v\left(N\left(j\right)\right)\right\|}_{2,\alpha }^{2}$ is the Gaussian‐weighted Euclidean distance between *N*(*i*) and *N*(*j*), α is the standard deviation of the Gaussian kernel, and *h* is the filtering parameter, which can affect the noise‐reduction performance.

As is well known, the ideal weighting function should output larger weights when the distance between pixels is smaller, and its output should decrease rapidly to zero with increasing distance. Therefore, an improved NLM algorithm was proposed, and its new weighting function can be expressed as

(4) $$w(i,j)=\left\{\begin{array}{ll} \exp\left(-\;\frac{D(i,j)}{h_{1}^{2}}\right)\cos\left(\frac{\pi D(i,j)}{2h_{2}}\right) & D(i,j)\leq h_{2}\\ 0 & D(i,j)>h_{2} \end{array}\right.,$$

where $D_{i,j}={\left\|v\left(N\left(i\right)\right)-v\left(N\left(j\right)\right)\right\|}_{2,\alpha }^{2}$, and *h*_1_ and *h*_2_ are filtering parameters.

### 2.3 Wavelet transform algorithm

From analysing and comparing five classical edge‐detection algorithms, a multi‐scale one based on B‐spline wavelets was used to obtain the boundaries of the structural features. The steps of image edge detection based on the wavelet transform were as follows.

Step 1 The original image was transformed into a grayscale one, and a wavelet transform was applied to calculate the high‐frequency detail component.

Step 2 The moduli of the wavelet‐transform coefficients were calculated. For a wavelet transform, usually taking $a=2^{j}\left(j\in Z\right)$, we obtain

(5) $$\left[\begin{array}{l} {\text{WT}}^{\left(1\right)}f\left(a,x,y\right)\\ {\text{WT}}^{\left(2\right)}f\left(a,x,y\right) \end{array}\right]=\text{WT}f\left(2^{j},x,y\right),$$

(6) modWTf2j,x,y=[|WT(1)f2j,x,y|2+|WT(2)f2j,x,y|2]12,

where $\;f\left(x,y\right)\in L^{2}\left(R^{2}\right)$.

Step 3 The amplitudes of the wavelet‐transform coefficients were calculated as

(7) $$A_{2^{j}}f\left(x,y\right)=\arctan\left(\frac{{\text{WT}}^{\left(2\right)}f\left(2^{j},x,y\right)}{{\text{WT}}^{\left(1\right)}f\left(2^{j},x,y\right)}\right).$$

**FIGURE 1:**
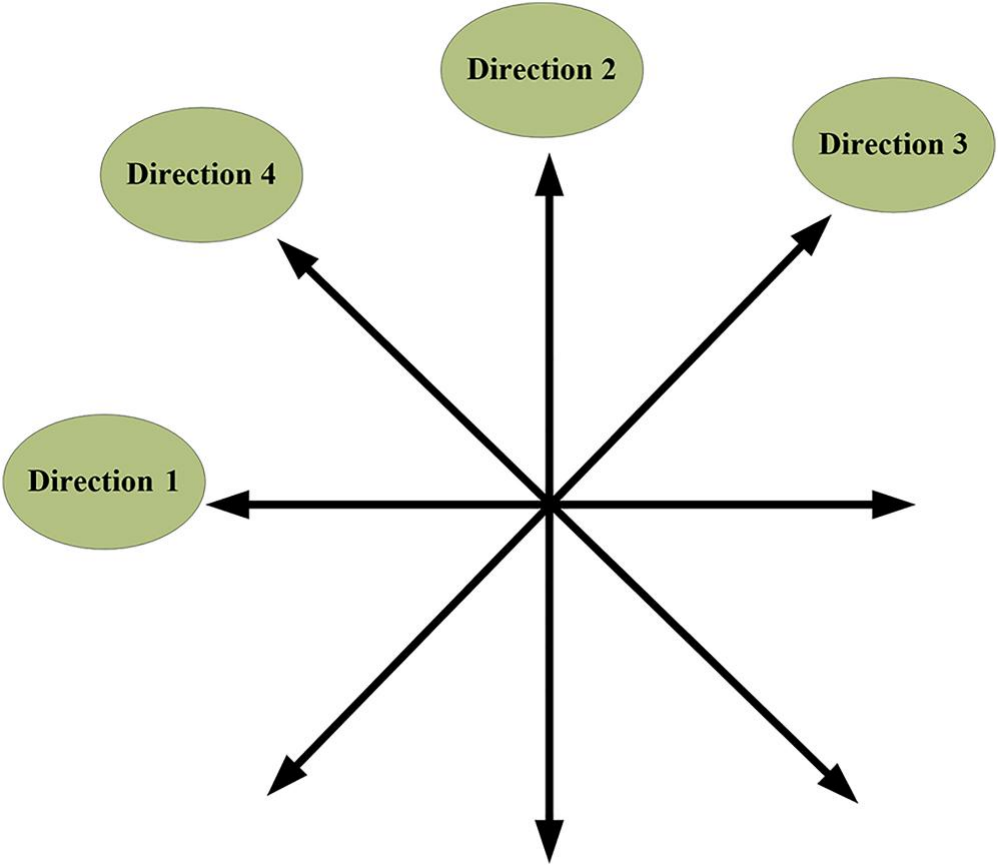
Angle pattern

Step 4 The local modulus maxima were obtained. The amplitude was divided into four directions (as shown in Figure 1): (i) 0° or 180°, (ii) 90° or 270°, (iii) 45° or 225°, and (iv) 135° or 315°.

Step 5 Each pixel was checked in turn to see whether it was the maximum value in the direction of the closest angle. If so, the gradient value was recorded; otherwise, the gradient value was set to zero.

Step 6 The initial edge image was processed by thresholding. A threshold value *λ* was selected, and the modulus values of all pixels whose modulus maximum value was less than *λ* were set to zero.

Step 7 The scale parameters of the wavelet transform were adjusted, and an edge‐detection image was output at each scale.

Step 8 The programme was run, and the image edges were output.

### 2.4 Test instruments and indentation methods

Nanoindentation has emerged as a leading technique for investigating small, thin, local, and specific regions of the nanomechanical properties of inhomogeneous biomaterials, and this allows for local probing at micro‐ and nanoscales [34, 35]. In the present study, nanoindentation experiments were performed in the central regions of the claws of *C. buqueti* Guer using a quantitative nanomechanical testing instrument (Nano Indenter G200; Agilent Technologies, Inc. USA) equipped with a Berkovich diamond indenter tip and a scanning probe microscope; a tip radius of 200 nm was chosen. The nanoindenter transducer with the indenter tip was calibrated before each indentation test, then a trapezoidal loading function was applied for the test with the following parameters: a maximum load of 3 mN, a loading rate of 50 µN/s, a holding time of 50 s, and a Poisson's ratio of 0.3; the thermal drift was less than 0.05 nm/s. The ambient temperature was 25°C and the humidity was 70%.

The Oliver–Pharr method was used to analyse the nanoindentation load–displacement data to determine the nanohardness (*H*) and elastic modulus (*E*_*r*_). The indentation *H* was calculated as

(8) $$H=\frac{P_{\max}}{A_{c}},$$

where *A*_*c*_ is the projected contact area of the indentation and *P*_max_ is the peak load. The reduced modulus *E*_*r*_ was calculated as

(9) $$E_{r}=\frac{\sqrt{\pi }S}{2\beta \sqrt{A_{c}}},$$

where *S* is the stiffness obtained by evaluating the slope of the curve fit at the onset of unloading, and *E*_*r*_ is the reduced elastic modulus.

The specimen modulus (*E*) is related to the reduced modulus as

(10) $$E_{r}={\left(\frac{1-v^{2}}{E}+\frac{1-{v_{i}}^{2}}{E_{i}}\right)}^{-1},$$

where v is the Poisson's ratio of the material; for the material comprising the indenter, its Poisson ratio was $v_{i}$  = 0.07 and its Young's modulus was $E_{i}$ = 1140 GPa.

## 3 RESULTS AND DISCUSSION

### 3.1 SEM imaging

**FIGURE 2:**
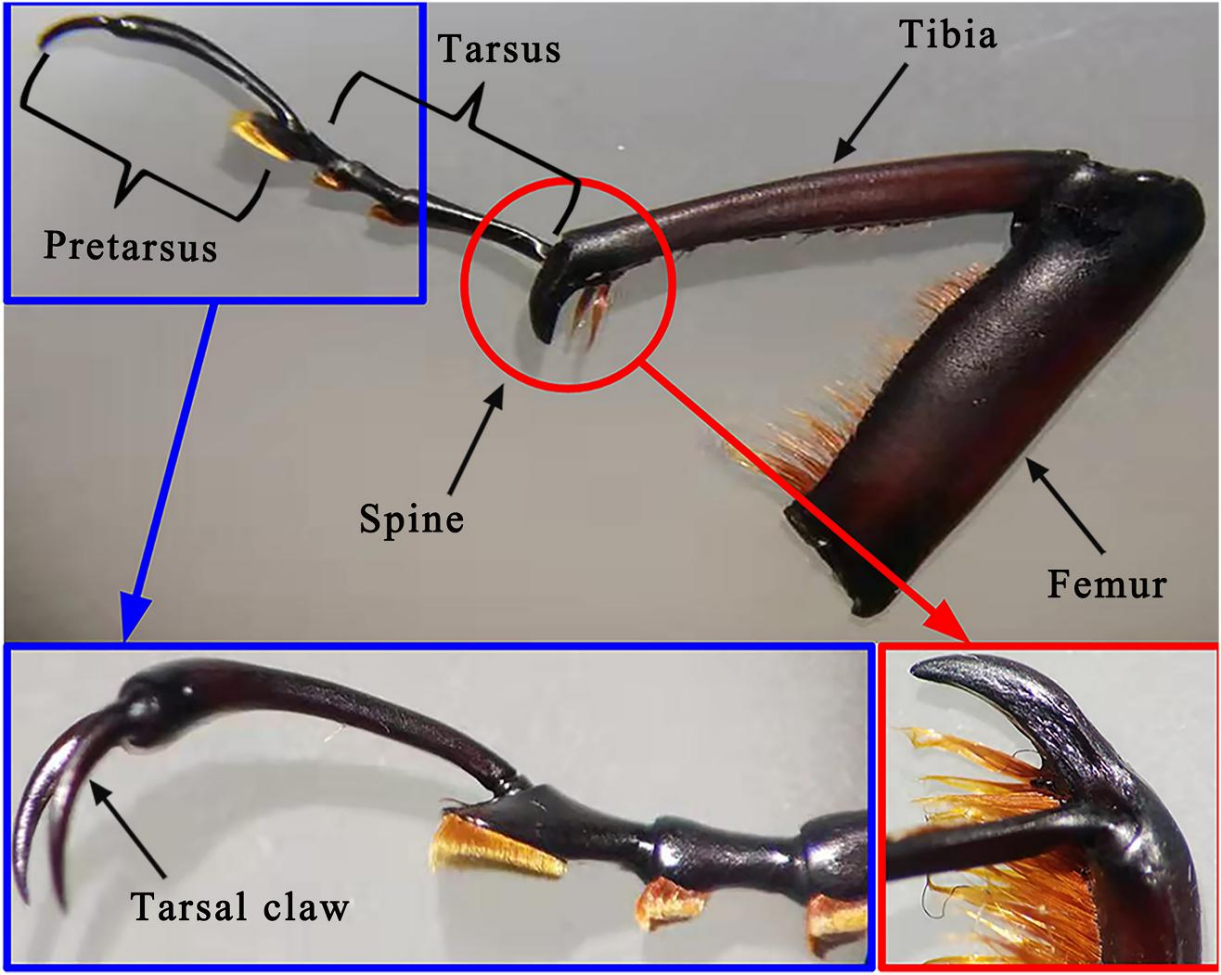
Structures of forefoot of *Cyrtotrachelus buqueti* Guer

SEM imaging was used to investigate the morphology of the claw surface, texture, and cross‐sectional structure. Figure 2 shows the structures of the weevil's forefoot, comprising the coxa, trochanter, femur, tibia, tarsus, and pretarsus. In *C. buqueti* Guer, the tarsal formation is equipped with a pair of terminal claws that are quite sharp and elongated, and the hook structure is adapted for movement on coarse or rough surfaces.

**FIGURE 3:**
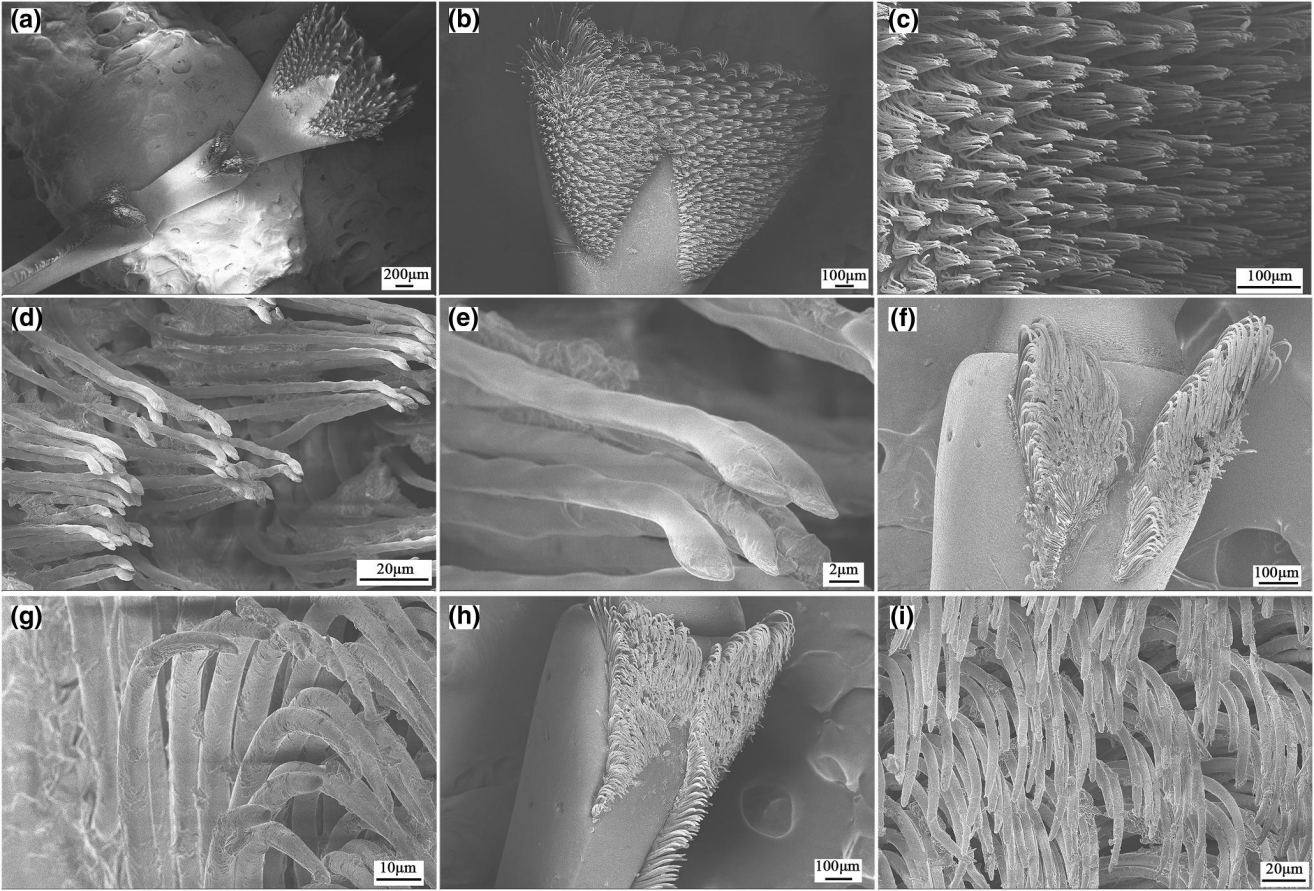
Functional morphology of tarsus of *Cyrtotrachelus buqueti* Guer: (a) tarsus; (b) first subsection of tarsus; (c) details in an enlarged view of a portion of (b); (d) details in an enlarged view of a portion of (c); (e) details in an enlarged view of a portion of (d); (f) the second subsection of tarsus; (g) setae in the second subsection; (h) the third subsection of tarsus; and (i) setae in the third subsection

**FIGURE 4:**
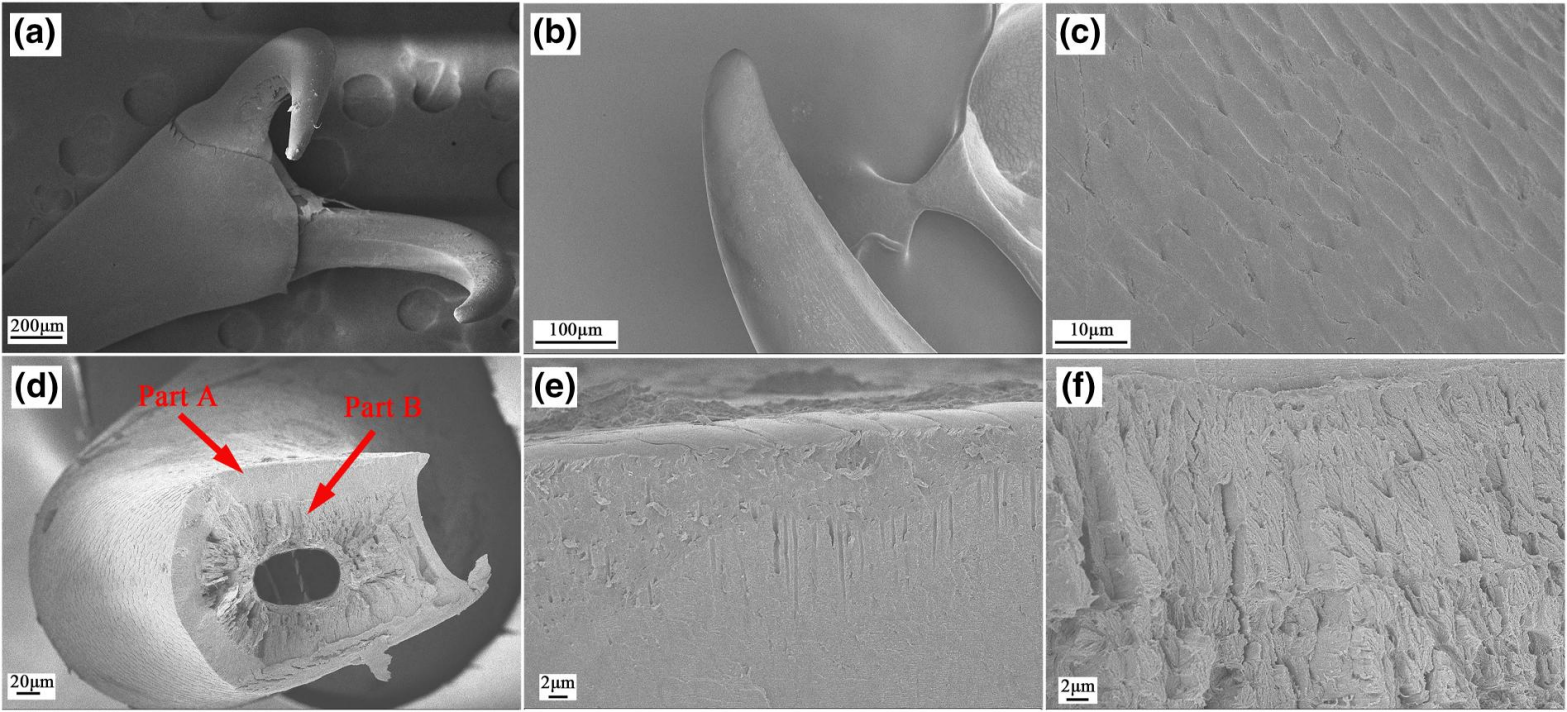
Functional morphology and ultrastructure of pretarsus: (a) claws; (b) lateral view of a claw; (c) surface of a claw; (d) longitudinal view of a claw showing a multilayer structure; enlarged views of surfaces of (e) part A and (f) part B as labelled in (d); (g) transverse view of a claw; and enlarged views of surfaces of (h) part C and (i) part D as labelled in (g)

The tarsus is the fifth segment of the thoracic foot in the weevil, and this has three subsections (Figure 3a). The first subsection of the tarsus is the largest, and it has a triangle‐like structure that bears the pretarsus (Figure 3b). It has a large number of cylindrical setae, and its end is conical. There are about 5–10 setae in a cluster, and the maximum diameter of the cylindrical setae is 2.436 ± 0.5 µm (*N* = 10), which was obtained by SEM measurements (Figure 3b–e). Additionally, the microstructures of the second and third subsections are basically rod shaped and also have many setae on the edges of both sides (Figure 3f–i). The pretarsus is at the end of the foot, and this bears the claws, which have scale‐like structures on their surfaces; they are hollow multilayered structures, and a typical cross section of a claw is shown in Figure 4d. The claw wall consists of two different structures, marked A and B in Figure 4d, and the details of these structures are shown in Figure 4e,f. From this analysis, we conclude that the adhesion force of the weevil comprises mainly the grip force of its claws and the van der Waals forces provided by its footpads, of which the grip force is primary. The claw structure comprises an exocuticle layer (exo in Figure 4g) and an endocuticle layer (endo in Figure 4g) with the latter being the thickest area. The exocuticle layer has a dense structure, while the endocuticle layer comprises fibres with different thickness that decreases in the radial direction. These special fibre layer structures make the claw highly light, strong, and stiff, as well as requiring minimal materials and conferring optimal mechanical properties. In the present study, we focussed mainly on the structure–function relationships and mechanical properties of the claws.

### 3.2 Mathematical model of claw

**FIGURE 5:**
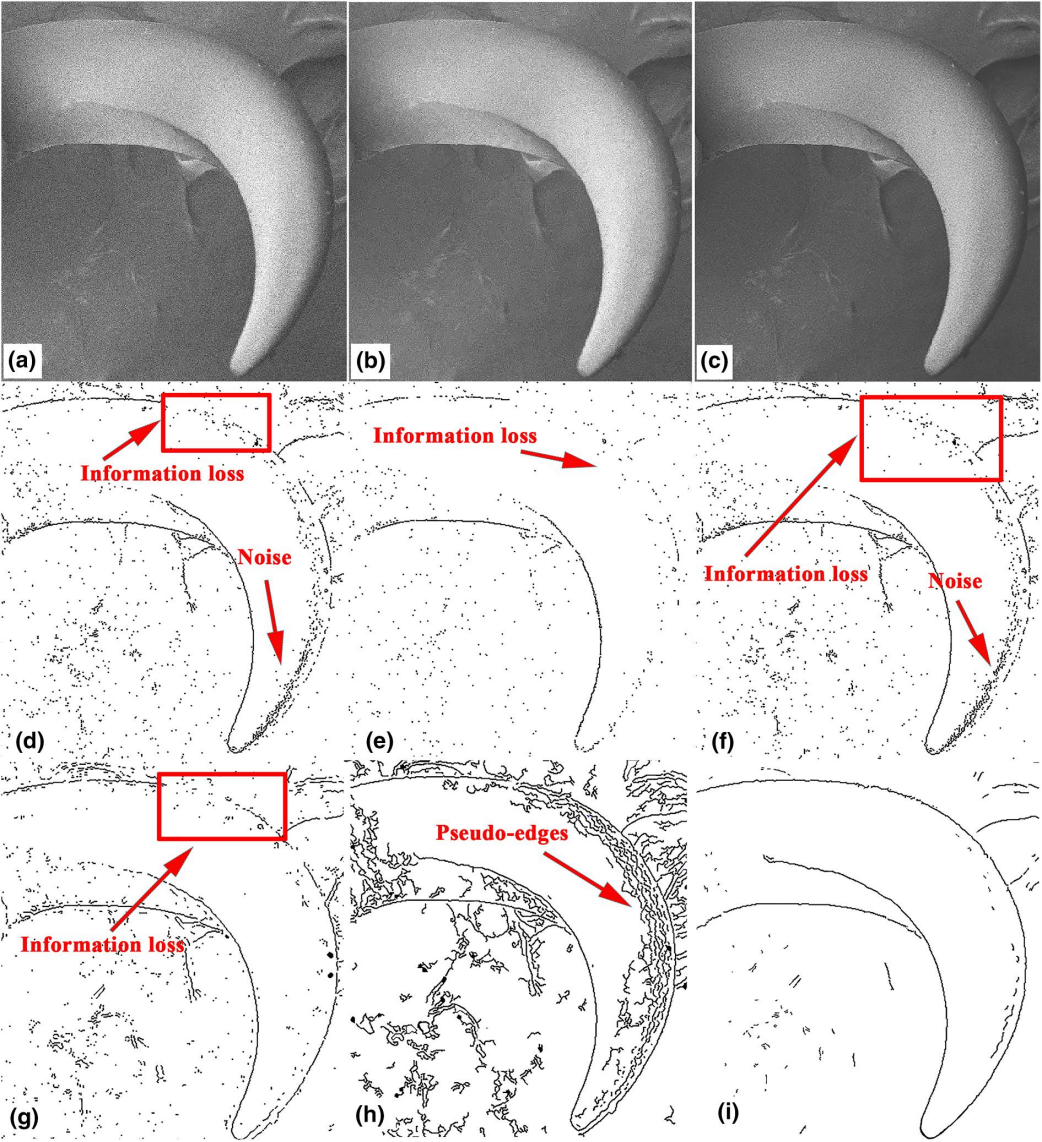
Claw of *Cyrtotrachelus buqueti* Guer: (a) original scanning electron microscopy (SEM) image of a claw of *C. buqueti* Guer; (b) image denoised using the original non‐local means (NLM) algorithm; (c) image denoised using the improved NLM algorithm; edge detection with (d) Sobel operator, (e) Roberts operator, (f) Prewitt operator, (g) Laplacian of Gaussian operator, (h) Canny operator, and (i) the multi‐scale edge‐detection algorithm based on B‐spline wavelets

Figure 5a shows a SEM image of a claw, and Figure 5b,c shows versions of this image denoised using the original and improved NLM algorithms, respectively. As can be seen, the denoised images have small errors when compared with the original image, and the effects of denoising using the two methods are distinct. The high peak signal‐to‐noise ratio (PSNR) and low mean‐squared error (MSE) in Table 1 indicate that the denoising results of the improved NLM algorithm are better than those of the original NLM algorithm. Note that the MSE is inversely proportional to the PSNR.

**TABLE 1:** Performance indices for original and improved NLM algorithms

Algorithm	Mean‐squared error	Peak signal‐to‐noise ratio
Original NLM	66.7758	29.8846
Improved NLM	62.2176	30.1917

Figures 5d–f show the effects of different edge‐detection algorithms applied to the claw. The Sobel, Roberts, and Prewitt operators resulted in poor edge extraction: neither the boundary curve is given completely, there are various missing edge features, and there are more miscellaneous points, so these operators are not good for detecting the claw edges. The Laplacian of Gaussian (LoG) and Canny operators detected the boundaries, but the LoG operator resulted in many discontinuous curves and false boundaries and a lack of edge information, and although the Canny operator limited the production of false boundaries to some extent, there were still many line segments and too much detailed information was retained. By contrast, the edge detection based on B‐spline wavelets is notably better: although there are some discontinuous line segments and miscellaneous points, the important boundary information is retained, the extraction of false boundaries has been reduced greatly, and the overall accuracy of boundary information extraction and recognition is higher.

A least‐squares method was used to fit the claw feature curves. This method minimises the sum of the squares of the differences between the original data points $y_{i}$ and the fitting data points $\overset{\wedge }{y_{i}}$ as described by

(11) $${\sum_{i=1}^{m}\left[h^{*}\left(x\right)-y_{i}\right]}^{2}=\min_{h\left(x\right)\in \phi }{\sum_{i=1}^{m}\left[h\left(x\right)-y_{i}\right]}^{2},$$

where ϕ represents a function class, h(x) is an arbitrary function in function class ϕ, and $h^{*}\left(x\right)$ is a function to find in function class ϕ.

The fitting accuracy is evaluated by the coefficient of determination *R* ^2^ as

(12) $$R^{2}=1-\frac{\text{SSE}}{\text{SST}},$$

where

(13) $$\text{SSE}={\sum_{i=1}^{n}\left(y_{i}-\overset{\wedge }{y_{i}}\right)}^{2},$$

(14) $$\text{SST}={\sum_{i=1}^{n}\left(y_{i}-\bar{y_{i}}\right)}^{2}.$$

**FIGURE 6:**
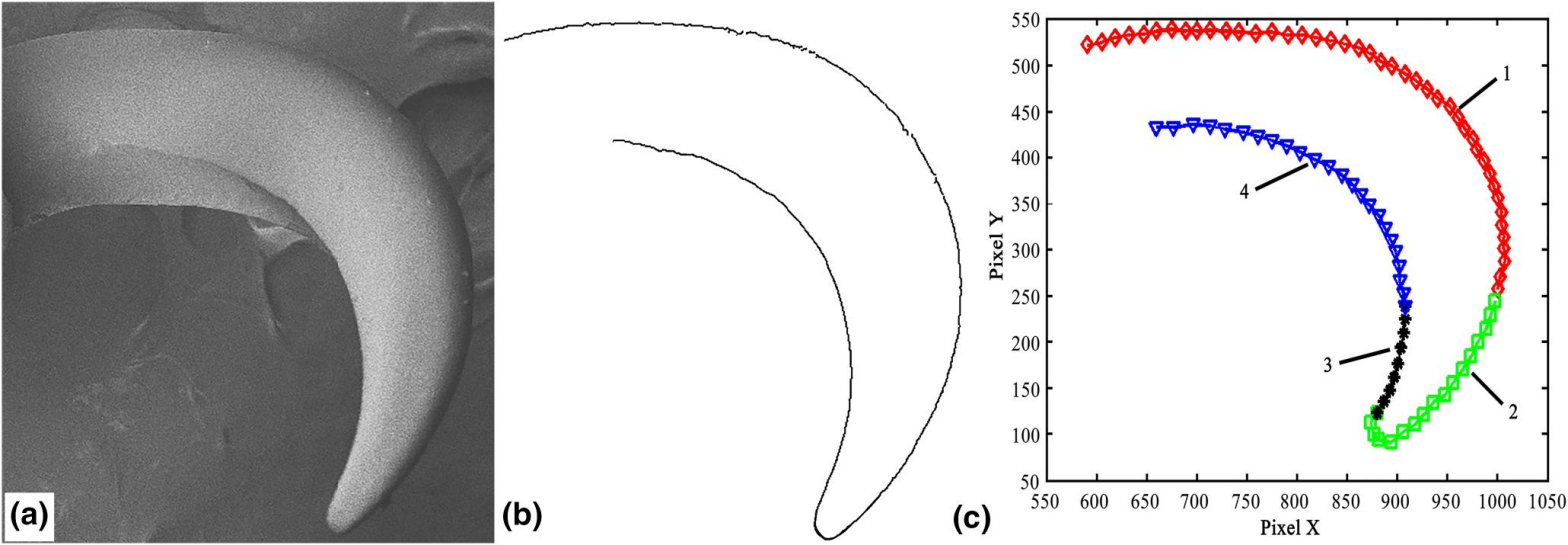
Feature boundary curves of claw: (a) SEM image of claw denoised using the improved NLM algorithm; (b) full boundary curves of claw; (c) plot showing the four curves into which the claw boundary was divided for fitting

Values of *R* ^2^ close to 1 indicate a very high fitting precision. For convenience of comparison, Figure 6a reproduces the denoised SEM image in Figure 5c, and the contour boundary curves extracted from this image are shown in Figure 6b.

To improve the fitting accuracy, the claw was divided into four characteristic curves that were fitted separately (Figure 6c). Four functions—a second‐order Gaussian function, a second‐order Fourier function, a second‐order polynomial function, and a third‐order polynomial function—were used to fit the geometrical characteristics of the claw, and Table 2 shows that the second‐order Fourier function had the highest fitting precision with *R* ^2^ values that are all nearly equal to 1.

**TABLE 2:** Values of judgement indices for fitting functions

Fitting function	Second‐order Gaussian function	Second‐order Fourier function	Second‐order polynomial function	Third‐order polynomial function
First fitting curve				
SSE	1.743e × 10^4^	1.388e × 10^4^	1.393e × 10^4^	1.897e × 10^4^
*R* ^2^	0.9491	0.9594	0.9009	0.9446
Second fitting curve				
SSE	852.5	547.5	748.4	719.9
*R* ^2^	0.9786	0.9863	0.9812	0.9819
Third fitting curve				
SSE	286.1	188.3	323	211.8
*R* ^2^	0.9782	0.9857	0.9754	0.9839
Fourth fitting curve				
SSE	1406	826.8	4536	1907
*R* ^2^	0.9853	0.9914	0.9526	0.9801

The Fourier function is defined as

(15) $$f\left(x\right)=a_{0}+\sum_{i=1}^{n}\left(a_{i}\;\cos\left(ixw\right)+b_{i}\;\sin\left(ixw\right)\right),$$

and Table 2 shows that the *R* ^2^ values for the second‐order Fourier function are each greater than 0.95, indicating that this function can achieve high accuracy. The fitting equation is given in Table 3.

**TABLE 3:** Equation for fitting claw feature curves

Equation coefficients	*f*(*x*) = *a*_0_ + *a*_1_cos (*xw*) + *b*_1_sin (*xw*) + *a*_2_cos (2*xw*) + *b*_2_sin (2*xw*)			
	First curve equation	Second curve equation	Third curve equation	Fourth curve equation
*a*_0_	−2.136e × 10^12^	216.4	3.167e × 10^9^	−1.942e × 10^13^
*a*_1_	2.847e × 10^12^	125.5	−2.394e × 10^9^	2.589e × 10^13^
*b*_1_	4.534e × 10^10^	−40.1	3.479e × 10^9^	−3.502e × 10^11^
*a*_2_	−7.115e × 10^11^	29.52	−3.773e × 10^8^	−6.47e × 10^12^
*b*_2_	−2.267e × 10^10^	4.189	−9.861e × 10^8^	1.751e × 10^11^
*w*	2.09e × 10^−5^	0.02373	−0.001086	−1.774e × 10^−5^
*R* ^2^	0.9594	0.9863	0.9866	0.9914
SSE	1.388e × 10^4^	547.5	175.7	826.8

**FIGURE 7:**
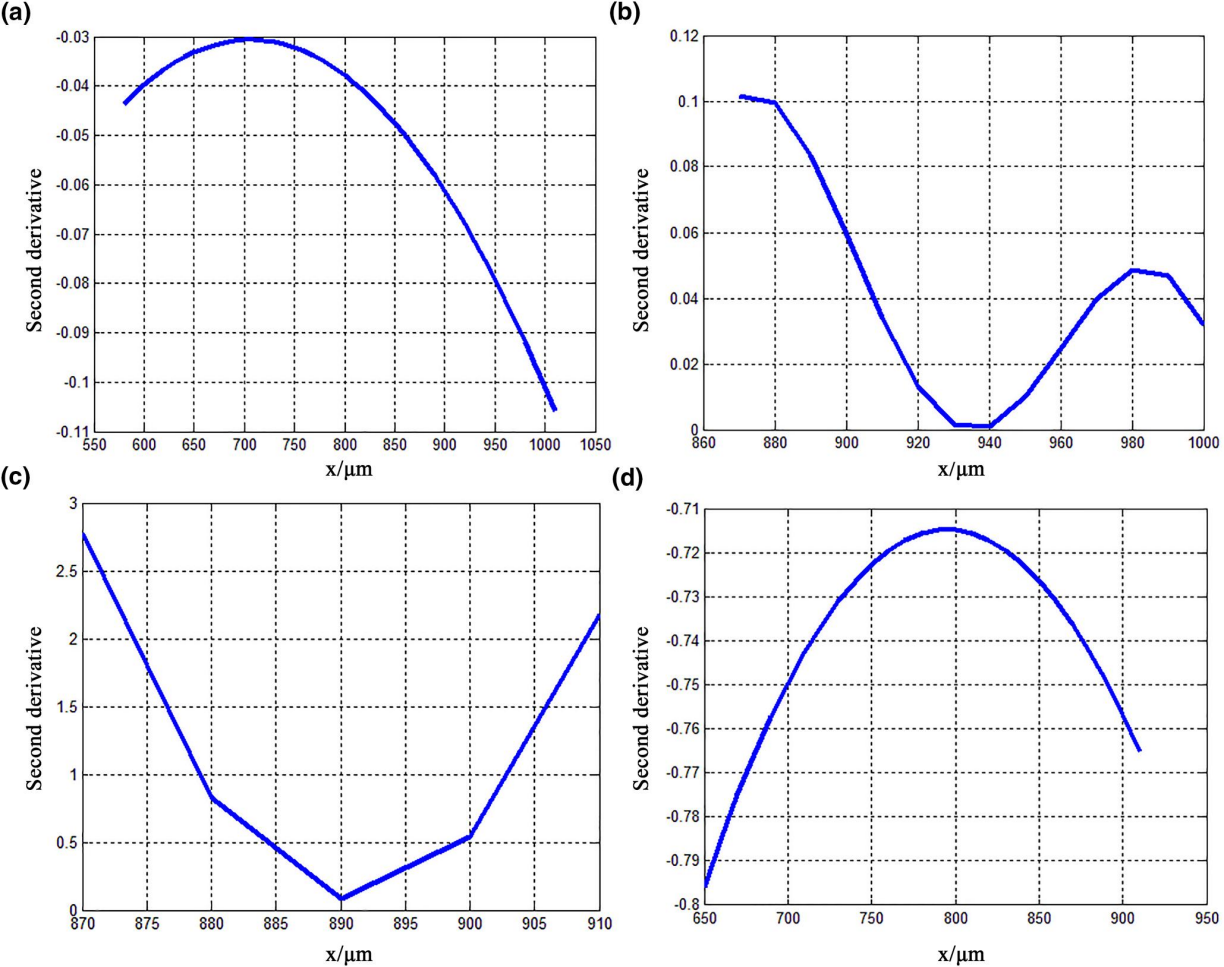
Second‐derivative variations of fitted curves for claw of *Cyrtotrachelus buqueti* Guer, showing (a) curve 1, (b) curve 2, (c) curve 3, and (d) curve 4

**FIGURE 8:**
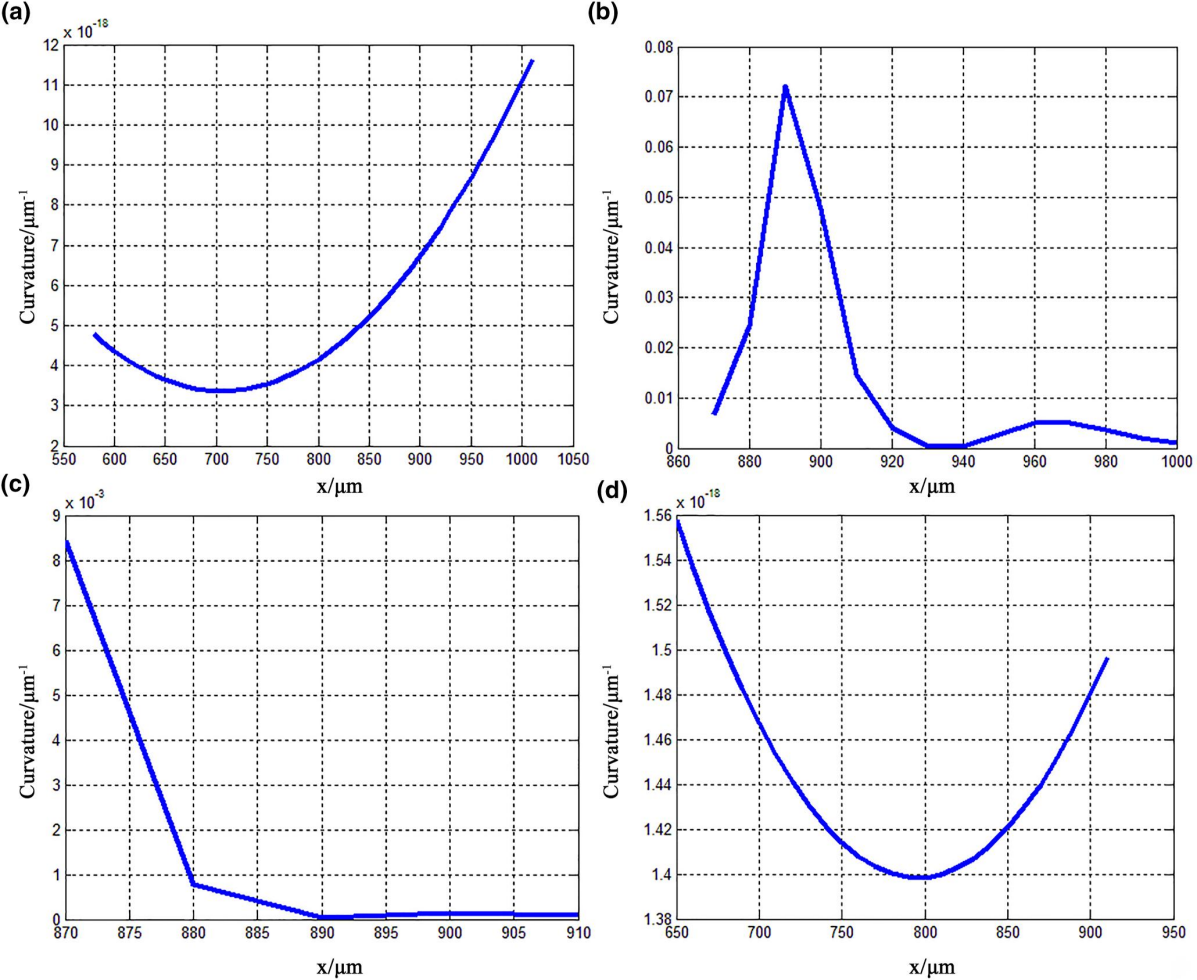
Curvature variations of fitted curves for claw of *Cyrtotrachelus buqueti* Guer, showing (a) curve 1, (b) curve 2, (c) curve 3, and (d) curve 4

According to the feature‐curve fitting equation, the curvature *K* can be calculated as

(16) $$K=\frac{\left|y^{\prime \prime }\right|}{{\left(1+y\prime^{2}\right)}^{\frac{3}{2}}},$$

where $y^{\prime }$ is the first derivative of the fitted curve and $y^{\prime \prime }$ is the second derivative. The second‐derivative and curvature variations for the claw were calculated using MATLAB, and the results are shown in Figures 7 and 8, respectively. Figure 7a shows that the second derivative of feature curve 1 is negative, indicating that it has a concave shape; this curvature decreases initially along the abscissa and then increases. As shown in Figure 8a, the curvature of curve 1 is very small, and its maximum value is less than 12 × 10^−18^ μm^−1^, which indicates that the curvature radius is larger, the results for the contour surface are smooth, and the changes are gentle; these geometric features offer notably reduced frictional resistance and help to avoid adhesion to bamboo. Figure 7b shows that the second derivative of feature curve 2 is positive, indicating that it has a convex shape. The curvature of feature curve 2 changes significantly and has two peak values; its values are the greatest of all the feature curves, and the maximum appears between 800 and 900 µm, which indicates that the curvature radius is small and the degree of curvature is great, resulting in the appearance of an arc shape (Figure 8b).

The curvature and variations of the claws of *C. buqueti* Guer help bamboo fibres to fall off their surfaces and enhance the adhesion force, and they reduce the stress concentration, thereby improving their structural strength. The second derivative of feature curve 3 is positive, indicating that the curve changes with a convex shape (Figure 7c). This curvature decreases, and the radius of curvature increases (Figure 8c). Finally, the second derivative of feature curve 4 is negative and shows a concave shape (Figure 7d). The curvature decreases again and the curvature radius increases rapidly, showing that the inner contour of the claw tends to be flat (Figure 8d). Therefore, it can be summarised that the outer contour curve of a claw of *C. buqueti* Guer moves from convex to concave, while the inner contour moves from concave to convex. Its curvature changes as ‘small–large–small’, and the degree of curvature follows a ‘flat–bending–flat’ pattern. These results indicate that this pattern is conductive to enhancing the adhesion force and improving the strength of the claw.

### 3.3 Adhesion mechanism

**FIGURE 9:**
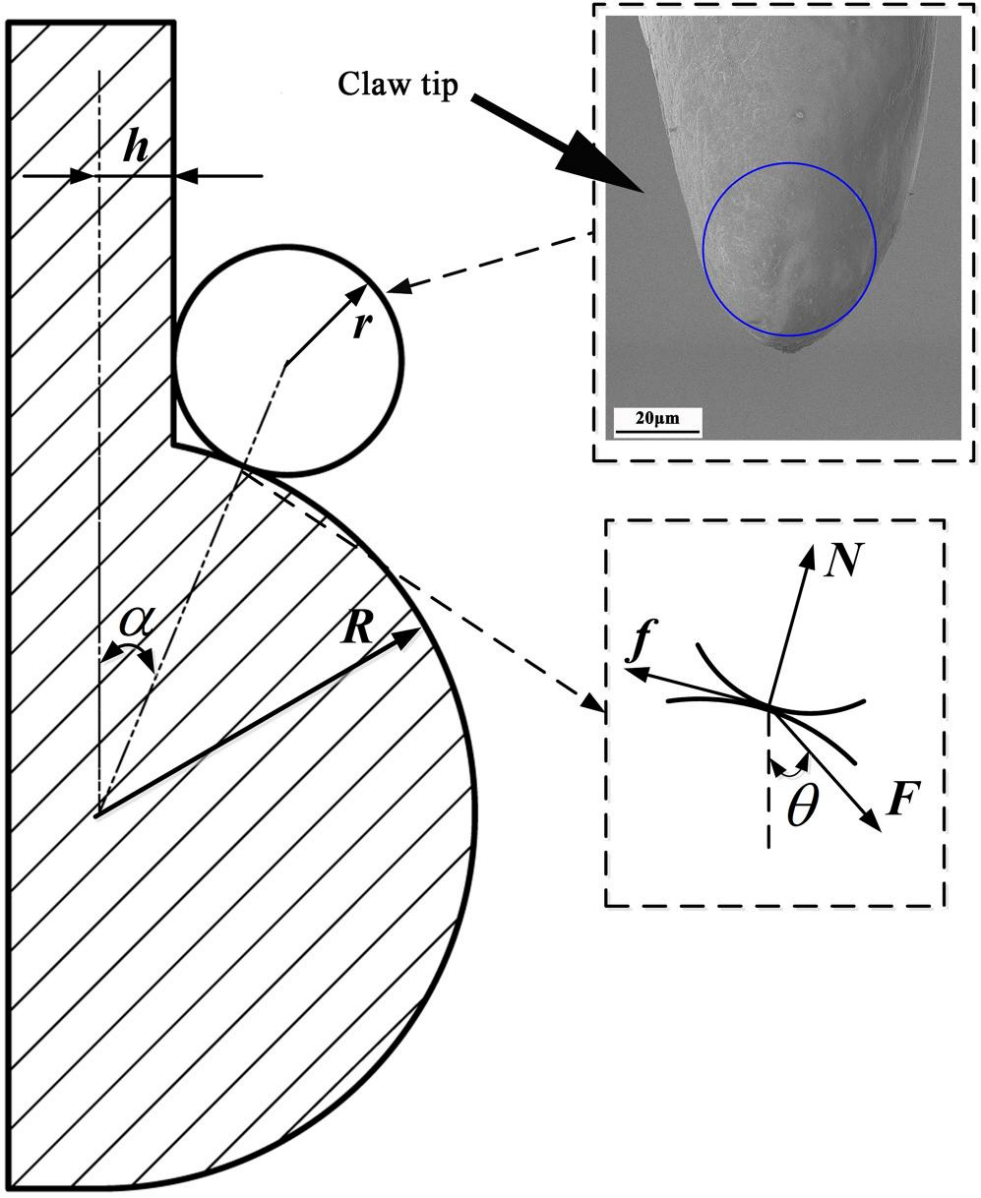
Adhesion model of claw on vertical surface (d>2(R+r))

The tip of a claw of *C. buqueti* Guer can be simplified as a circle, and its radius is set as *r* and the grasping object particle radius as *R*. Because of the uneven particle distribution on the grasping surface, the grasping form of the claw is influenced by the distance *d* between two particles. Therefore, herein, we discuss the situation for different values of *d*. We assume that when *d* is larger, that is, d>2(R+r), the gripping model of the claw is shown in Figure 9, where the model has been reduced to the contact between two spherical surfaces. In Figure 9, *F* is the driving force provided by the claw (including gravity), α is the pressure angle, that is, the contact angle between the hook claw and the wall, *N* is the supporting force of the particle on the claw, *f* is tangential friction force of the claw tip along the wall, θ is the load angle, and *μ* is the friction coefficient.

The principles of mechanics give the following equilibrium relationships:

(17) $$\left\{\begin{array}{l} F\;\sin\;\theta =F_{f}\;\cos\;\alpha ‐N\;\sin\;\alpha \\ F\;\cos\;\theta =N\;\cos\;\alpha +F_{f}\;\sin\;\alpha \\ F_{f}=\mu N \end{array}\right.,$$

which in turn give

(18) $$\frac{F\;\sin\;\theta }{F\;\cos\;\theta }=\frac{\mu \;\cos\;\alpha ‐\sin\;\alpha }{\cos\;\alpha +\mu \;\sin\;\alpha },$$

according to which the load angle can be expressed via

(19) $$\tan\;\theta =\frac{\mu -\tan\;\alpha }{\mu \;\tan\;\alpha -1}$$

as

(20) θ=arctanμ−α.

Also, Figure 9 gives the relationship

**FIGURE 10:**
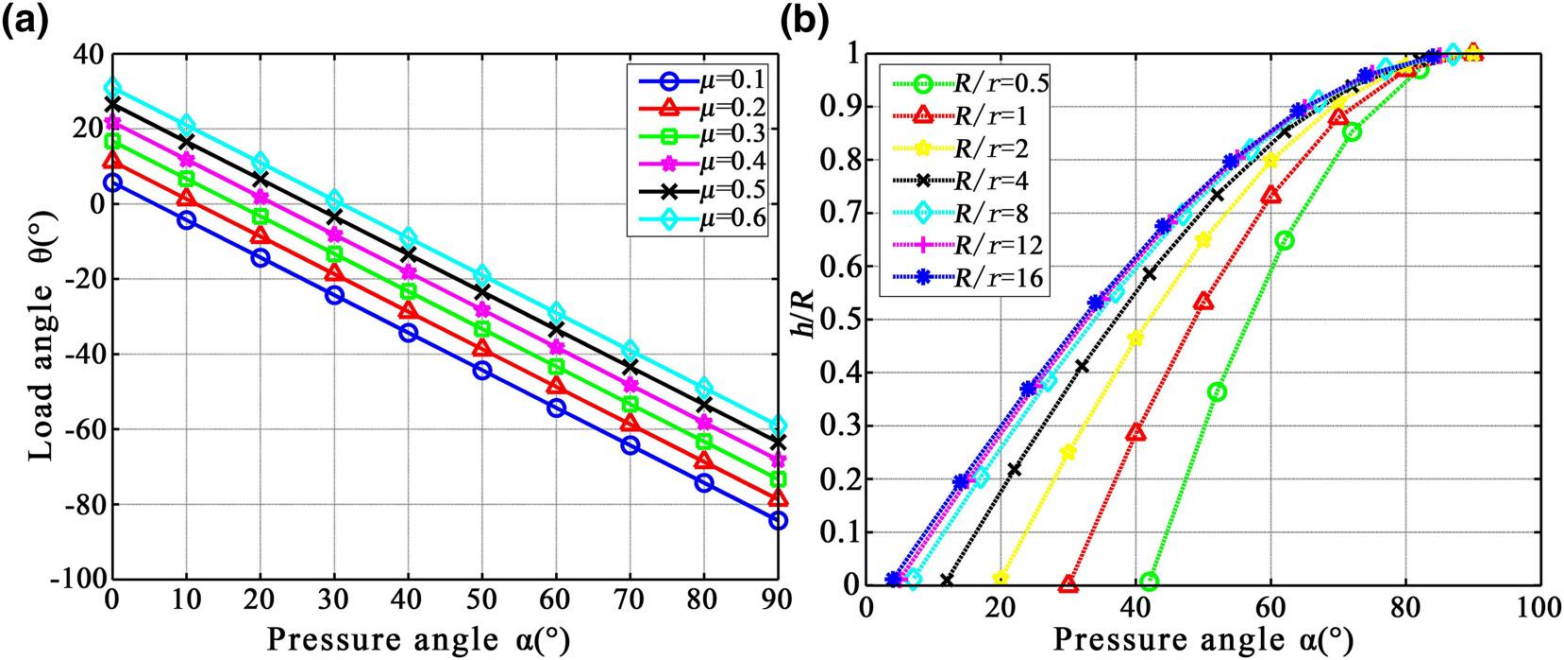
(a) Relationship among load angle θ, pressure angle α, and friction coefficient μ. (b) Relationship among claw tip radius r, particle radius R, and pressure angle α

(21) sinα=r+hr+R=rR+hRrR+1,

and Equations (20) and (21) give the relationships among (i) the load angle θ, the pressure angle α, and the friction coefficient *μ* and (ii) the claw tip radius *r*, the particle radius *R*, and the pressure angle α, as shown in Figure 10.

Figure 10a indicates that when the friction coefficient *μ* is constant, the load angle θ decreases with increasing pressure angle α. On the other hand, when the pressure angle α is constant, the friction coefficient *μ* increases, and the load angle θ that the claw tip bears is also larger, which is conducive to grasping by the weevil claw. However, when a pressure angle α=arctanμ, the load angle θ is zero at this time, and the claw cannot self‐lock and so disengages. Figure 10b shows that the larger the value of *R*/*r* (i.e., when the particle radius exceeds that at the end of the claw), (i) the smaller the ratio *h*/*R* of embedded depth to particle radius, (ii) the smaller the pressure angle, and (iii) the easier it is for the claw to attach to the surface, thereby satisfying the conditions for attachment. However, if the embedded depth *h* is too large, then when the limit position *h* = *R* is reached, the tip size of the claw no longer meets the adhesion conditions and the claw cannot adhere to the surface.

These results provide an important basis for developing bionic wall‐climbing robots. For example, for contact surfaces with a certain coefficient of friction, the gripping ability of a robot can be improved by reducing the pressure angle α (the spherical radius *r* of the claw tip). Similarly, if the radius *r* of the claw tip of a robot is limited by either the material or existing processing technology, then increasing the friction coefficient *μ* between the claw and the contact surface can also effectively improve the gripping ability.

**FIGURE 11:**
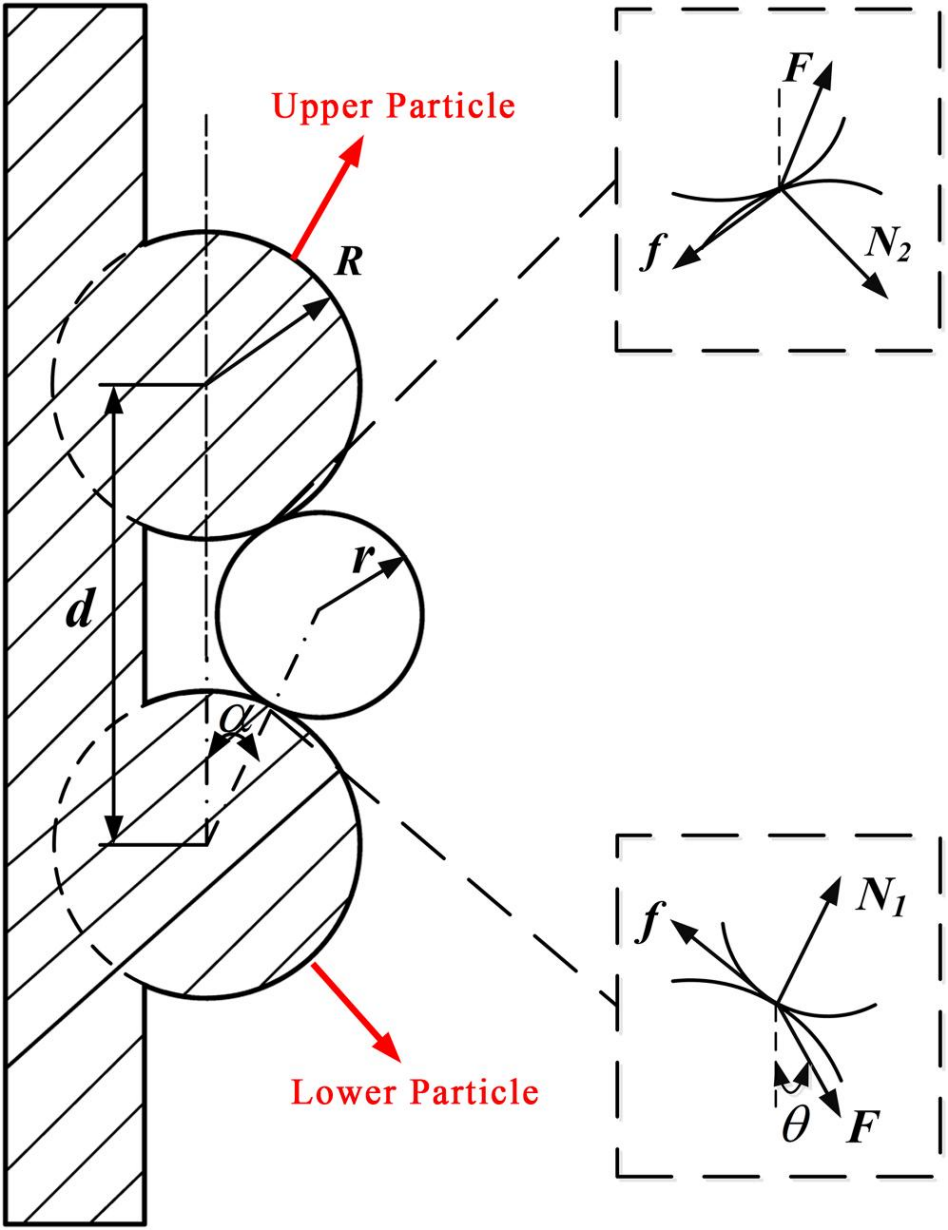
Adhesion model of claw on vertical surface (d<2(R+r))

However, if the distance *d* between particles is small, that is, d<2(R+r), then the claw adhesion model becomes as shown in Figure 11, which gives

(22) $${\left(R+r\right)}^{2}=d^{2}+{\left(R+r\right)}^{2}-2d\left(R+r\right)\cos\;\alpha ,$$

that is,

(23) cosα=d2(R+r),

(24) α=arccosd2(R+r)=arccosdR2(1+rR).

**FIGURE 12:**
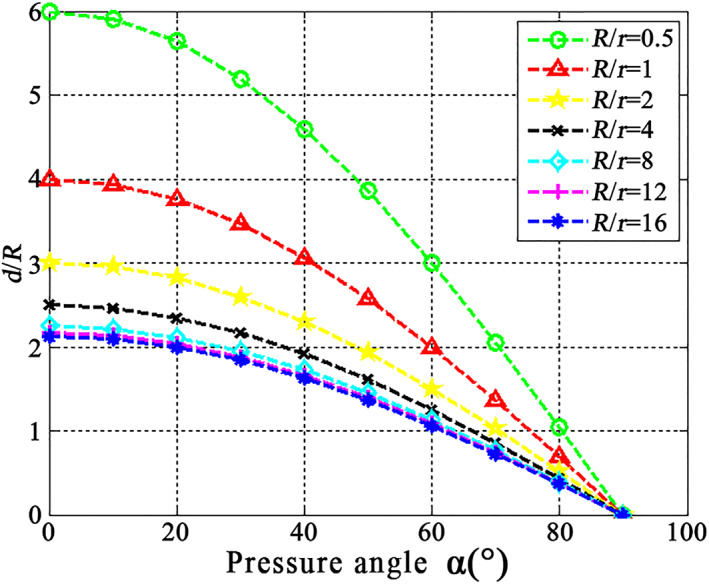
Relationship among interparticle distance *d*, particle radius *R*, and claw radius *r*

From Equation (24), we used MATLAB to plot the relationship among the interparticle distance *d*, the particle radius *R*, and the claw radius *r* as shown in Figure 12.

Figure 12 shows that for constant *R/r*, the pressure angle α increases with decreasing *D*/*R*, and for constant *D/R*, the pressure angle α decreases with increasing *R/r*. Therefore, reducing the claw radius *r* can also reduce the pressure angle α and increase the adhesion force. If the interparticle distance *d* is smaller, so the pressure angle α increases, resulting in reduced adhesion.

Similarly, simplifying the driving force *F* and the supporting forces *N*_1_ and *N*_2_ and friction forces *F*_*f*1_ and *F*_*f*2_ provided by the weevil in the horizontal and vertical directions, respectively, yield the following equations:

(25) $$\left\{\begin{array}{l} F_{p}=F\;\sin\;\theta \\ F_{v}=F\;\cos\;\theta \\ F_{f1}\;\cos\;\alpha =N_{1}\;\sin\;\alpha +F_{p1}\\ F_{f1}\;\sin\;\alpha +N_{1}\;\cos\;\alpha =F_{v1}\\ F_{f2}\;\cos\;\alpha =N_{2}\;\sin\;\alpha +F_{p2}\\ F_{f2}\;\sin\;\alpha +N_{2}\;\cos\;\alpha =F_{v2} \end{array}\right.$$

where

$$\left\{\begin{array}{l} F_{f1}=\mu N_{1}\\ F_{f2}=\mu N_{2} \end{array}.\right.$$

where *F*_*p*_ and *F*_*v*_ are the components of the driving force *F* in the horizontal and vertical directions, respectively; *F*_*p*1_ and *F*_*v*1_ are the force components of the lower particle contact point in the horizontal and vertical directions, respectively; *F*_*p*2_ and *F*_*v*2_ are the force components of the upper particle contact point in the horizontal and vertical directions, respectively; *N*_1_ and *N*_2_ are the supporting forces of the lower and upper particles, respectively; and *μ* is the friction coefficient.

Simplifying the Equation (25) gives the relationship among *N*_1_, *N*_2_, α, and θ as

(26) $$F_{p}=\left(N_{1}-N_{2}\right)\left(\mu \;\cos\;\alpha -\sin\;\alpha \right),$$

(27) $$F_{v}=\left(N_{1}+N_{2}\right)\left(\mu \;\sin\;\alpha +\cos\;\alpha \right).$$

Letting $k=\frac{1}{m}=\frac{N_{1}}{N_{2}}$ gives

(28) $$k=\frac{1}{m}=\frac{N_{1}}{N_{2}}=\frac{\tan\;\alpha -\mu -\mu \;\tan\;\alpha \;\tan\;\theta -\tan\;\theta }{\tan\;\alpha -\mu +\mu \;\tan\;\alpha \;\tan\;\theta +\tan\;\theta },$$

(29) $$N_{1}=\frac{F_{p}}{\left(1-m\right)\left(\mu \;\cos\;\alpha -\sin\;\alpha \right)},$$

(30) $$N_{2}=\frac{N_{1}}{k}.$$

Similarly, *F*_*p*_ and *F*_*v*_ can be rewritten as

(31) $$F_{p}=\left(1-m\right)\left(\mu \;\cos\;\alpha -\sin\;\alpha \right)N_{1},$$

(32) $$F_{v}=\left(1+m\right)\left(\mu \;\sin\;\alpha +\cos\;\alpha \right)N_{1}.$$

**FIGURE 13:**
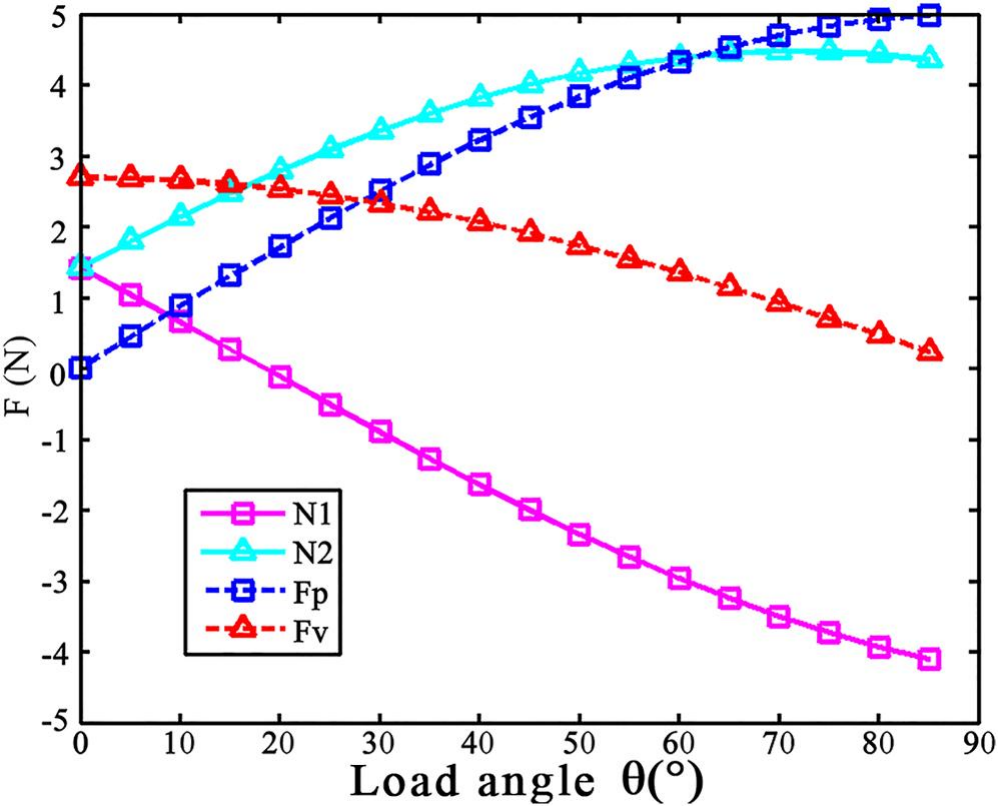
Relationship among *θ*, *N*_1_, *N*_2_, *F*_*p*_, and *F*_*v*_

To facilitate quantitative analysis, we set the pressure angle α to 45°, the friction coefficient *μ* to 0.5, and the grip force provided by the claw tip of the weevil to 3 N. Figure 13 shows the relationship among *θ*, *N*_1_, *N*_2_, *F*_*p*_, and *F*_*v*_. When *θ* = 0°, the claw is embedded vertically downward; there is no force component in the horizontal direction, and *N*_1_ and *N*_2_ are equal. With gradually increasing *θ*, *N*_2_ keeps increasing, and *N*_1_ first drops to zero and then increases in the reverse direction opposite to *N*_2_. We refer to the value of *θ* at which *N*_1_ = 0 as the limit value, that is, when *θ* is increased gradually to ∼15°, the claw acts on only one particle. When the angle exceeds this, the centre of gravity of the claw acts on the upper particle, and the upper particle is the main action point. *F*_*p*_ increases continuously with increasing *θ*, and *F*_*v*_ decreases with increasing θ, when *θ* equals to 90°; at this time, the vertical direction of the force *F*_*v*_ = 0, cannot be grasped attached. When the load angle *θ* = 60°, the resultant of the horizontal force *F*_*p*_ and the vertical force *F*_*v*_ is the largest, whereupon the gripping force is the strongest. Gripping experiments reported in the literature show that the gripping performance is best when the gripping load angle *θ* is between 45° and 60°, whereupon the adhesion force is stronger [36]; this agrees with the present findings.

### 3.4 Nanoindenter tests

As is well known, water content has a significant influence on the Young's modulus and hardness of the cuticle of a claw [37, 38]. To account for this, we also tested wet samples to investigate the differences in the mechanical properties when compared with the dry samples; here, ‘dry’ refers to samples that have been dried naturally for at least a month, and ‘wet’ refers to samples that have been immersed in water for an hour. To limit the water loss, the experiment was performed as soon as the samples were removed from the water.

**FIGURE 14:**
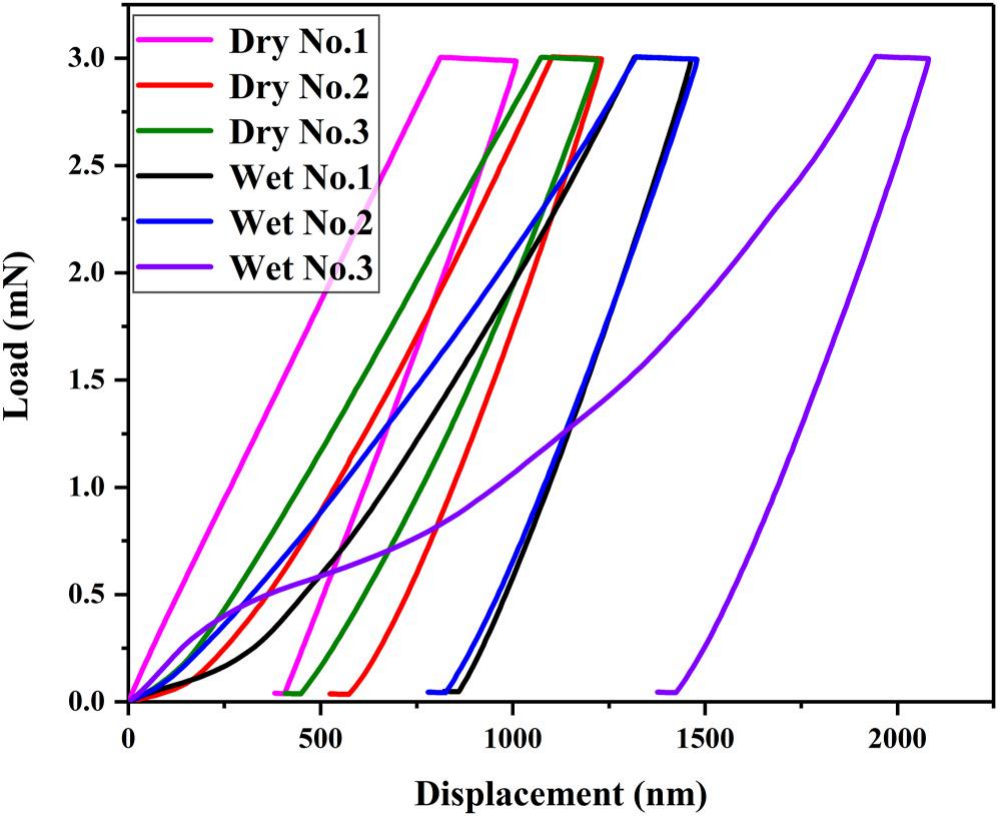
Resulting load–displacement curves for the claw of *Cyrtotrachelus buqueti* Guer

Representative load–displacement curves obtained for dry and wet samples are shown in Figure 14. The typical shape of the load–displacement curves indicates that the claw exhibits elastic–plastic behaviour. It is observed that all the loading–unloading curves are smooth. The hardness and modulus for the dry samples were 0.197 ± 0.074 and 1.105 ± 0.197 GPa, respectively, and the respective values for the wet samples were 0.071 ± 0.030 and 0.693 ± 0.163 GPa. The hardness and modulus of the dry samples were roughly 2.77 and 1.59 times those of the wet samples, respectively. These results concur with the understanding that the presence of water in the claw can significantly decrease its hardness and modulus. This is critical for analysing the mechanical behaviour of the claw as it undergoes deformation and providing a biological template for a climbing robot.

## 4 CONCLUSIONS

In this study, we analysed the mechanical performance of the claw structure in *C. buqueti* Guer and the relationship between its function and morphology, and we draw the following conclusions from the results obtained.

The morphology of the pretarsus, which bears the claw in the forefoot, was investigated by SEM. The SEM images showed that the pretarsus has three subsections, which have many setae and provide adhesion through van der Waals forces. The claw is a hollow multilayered structure, and its cross section indicates that it consists of different structural layers that provide the main grip force.

The NLM and improved NLM algorithms were used to reduce the noise and improve the quality of a SEM image of a claw. The results showed that the improved NLM algorithm gives better results than the original NLM algorithm. Additionally, the proposed edge‐detection algorithm was found to be more accurate than five other classical edge‐detection algorithms in extracting the boundary of the structural features.

Structure–function relationships were studied using a mathematical model. A least‐squares method was used to fit the contour boundary curves of the claw. The results indicated that a varied curvature pattern cannot only enhance the adhesion force but also improve the structural strength of a claw by reducing stress concentrations.

Adhesion models of the claw were established. The results shown that for contact surfaces with a certain coefficient of friction, the gripping ability can be improved by reducing the pressure angle α. Similarly, if the radius *r* of the claw tip of a robot is limited by either the material or existing processing technology, then increasing the friction coefficient *μ* between the claw and the contact surface can also effectively improve the gripping ability.

The hardness and modulus for dry claws were found to be 0.197 ± 0.074 and 1.105 ± 0.197 GPa, respectively, and the respective values for wet claws were 0.071 ± 0.030 and 0.693 ± 0.163 GPa. The presence of water was found to cause significant changes in hardness and modulus.

## CONFLICT OF INTEREST

The authors declare that there is no conflict of interests regarding the publication of this article.

## Data Availability

Author elects not to share the data.
